# Agent-Based Intelligent Interface for Wheelchair Movement Control

**DOI:** 10.3390/s18051511

**Published:** 2018-05-11

**Authors:** Alberto L. Barriuso, Javier Pérez-Marcos, Diego M. Jiménez-Bravo, Gabriel Villarrubia González, Juan F. De Paz

**Affiliations:** BISITE Digital Innovation Hub., University of Salamanca, Edificio Multiusos I+D+i, C/Espejo SN, 37007 Salamanca, Spain; jpmarcos@usal.es (J.P.-M.); dmjimenez@usal.es (D.M.J.-B.); gvg@usal.es (G.V.G.); fcofds@usal.es (J.F.D.P.)

**Keywords:** wheelchair control, multi-agent systems, disabled persons, control systems, human-computer interfaces

## Abstract

People who suffer from any kind of motor difficulty face serious complications to autonomously move in their daily lives. However, a growing number research projects which propose different powered wheelchairs control systems are arising. Despite of the interest of the research community in the area, there is no platform that allows an easy integration of various control methods that make use of heterogeneous sensors and computationally demanding algorithms. In this work, an architecture based on virtual organizations of agents is proposed that makes use of a flexible and scalable communication protocol that allows the deployment of embedded agents in computationally limited devices. In order to validate the proper functioning of the proposed system, it has been integrated into a conventional wheelchair and a set of alternative control interfaces have been developed and deployed, including a portable electroencephalography system, a voice interface or as specifically designed smartphone application. A set of tests were conducted to test both the platform adequacy and the accuracy and ease of use of the proposed control systems yielding positive results that can be useful in further wheelchair interfaces design and implementation.

## 1. Introduction

It is well known by all the problem that people with motor disabilities face to autonomously move in their daily lives. In the case of quadriplegic people who use wheelchairs, this problem is even more serious. According to the World Health Organization, around 10% of the world’s population suffers some type of disability (either physical or mental), of which around 10% need a wheelchair to move [1]. In Europe, according to a report published by the European Commission, the number of people with disabilities expanses to 38 million (16% of the population) and by 2050 it is estimated that 30% of the population will be over 65 years [2], so disabilities derived from aging will increase significantly.

Fortunately, society is increasingly aware of the challenge of integrating disabled people, especially in the field of mobility. As a consequence of this collective consciousness, different initiatives arise from large organizations. One of the most relevant initiatives is the declaration by the United Nations (UN) of the Standard Rules on the Equalization of Opportunities for Persons with Disabilities [3], which helped to put this problem in the spotlight, leading the way to the creation of other new initiatives in the field of the integration of people with disabilities. We can find some examples in European Disability Strategy 2010–2020: A Renewed Commitment to a Barrier-Free Europe [4] within the European Union or the Disability Integration Act of 2015 [5] in the United States.

The use of technological solutions in the field of health has significantly helped to improve our quality of life. The field of mobility is not oblivious to these improvements: since the appearance of the first motorized electric wheelchair developed by George Klein in the National Research Council (NRC) in the 50s [6], the dependence of people with motor disability has decreased noticeably thanks to the advances in this technology area. From this initial model, numerous improvements have been incorporated, such as the use of micro-processors for the improvement of their control and manoeuvrability, improvements in the autonomy of the chairs, the incorporation of suspensions and tilting seats, the appearance of new control interfaces, or even chairs that allow to go up and down stairs autonomously [7].

Despite the great advances that have been achieved, the wide variety of motor disabilities and degrees of disability affecting wheelchair users, make traditional wheelchair control devices not suitable for all of them. However, great progress is being made in applying artificial intelligence techniques to the control of motorized wheelchairs, adapting control systems to different disabilities. We can find several examples in previous works such as [8] where different control alternatives are offered for people with quadriplegia by using ocular movements or voice commands; [9] where ultrasonic sensors data is used in combination with fuzzy logic to implement an obstacle avoidance system, improving the navigation in confined areas; [10] where Force Sensing Resistors are used in a head-movement based controlled interface; or [11] where a hand gesture based control is implemented by using inertial measurement unit and electromyography sensors. These are some of the many examples in the current literature where we can see how the control systems are increasingly improving through the combination of machine learning techniques and the data collected from different sensors.

The growing number of wheelchair control alternatives and the increase of the computational load necessary to be able to deploy these control systems in combination of the sensors data acquisition process, makes it necessary to investigate in platforms that favour the rapid integration of diverse control systems on different wheelchair hardware platforms, so that they are adapted to the specific needs of each case. In this way, it will be possible to favour and expedite the research in new control systems and improve the adequacy of the use of a wheelchair to the highest number of users with mobility problems, regardless of the degree of disability that they present in order to favour their social integration.

With this in mind, this paper presents a platform based on virtual organizations (VO) of agents that enables the rapid integration of different human-machine interfaces in a conventional motorized wheelchair. For the successful achievement of this work, a hardware interface has been designed. This hardware interface acts as a bridge between the physical layer and the final applications and control systems that have been developed for users. This hardware interface allows to control the wheelchair’s motors, using a protocol compatible with USB or Bluetooth technologies. The different control systems that have been developed in this work for the possible users of the platform are: control based on voice controls, three different control possibilities based on the use of an smartphone (two of them using the touchscreen and one using the smartphone accelerometer value) and finally and as the main novelty in terms of control, the incorporation of an electroencephalogram (EEG) device, which allows detecting the movement that the user wants to make through the analysis and processing of the electrophysiological signals produced by the brain with machine learning techniques. The deployment and integration of this set of control systems in a conventional wheelchair has allowed to experiment the proper functioning of the proposed platform. Additionally, a control software that allows interacting with the user by collecting information on the state of the chair (battery status, speed, temperature, obstacle proximity sensors, chair autonomy, etc.) has been developed. The different types of control that have been integrated into our proposal aim to make our solution accessible to the greatest number of people, thus favouring the autonomy of people with mobility problems.

The document is structured as follows: Section 2 gives a brief review of the state of the art and related projects, Section 3 describes the proposed architecture, in Section 4 the results of the case study are presented, which will serve to evaluate the system and finally the conclusions are drawn in Section 5. Additionally, Appendix A has been included with the hardware design schemes used in this work.

## 2. Background

Since the advent of microprocessors, there have been significant improvements in the control interfaces for motorized wheelchairs [12]. A proof of this fact is the wide variety of options that are currently offered by wheelchair manufacturers. Within these control interfaces, two types are clearly differentiated: proportional controls and non-proportional controls [13].

Proportional controls are those that allow simultaneous control of speed and direction. The most common proportional control interface is the joystick. Besides of the traditional joysticks, there is a wide variety of joysticks with specific characteristics, such as sensitive joysticks, which allow to perform high precision movements by using few physical strength or sealed joysticks, which are resistant to liquids, of small size and it is also necessary to use less physical strength. In addition to joysticks, some of the most widespread proportional control devices are the touch pad, a control based on touch, very similar to the one used in laptops or tilt sensors, with which users perform small movements to choose the direction where they want to move. Table 1 shows a compilation of proportional control systems for wheelchairs that can be found on the market today.

As for the non-proportional controls, they allow the user of the wheelchair to control only the direction of movement, not the speed. Parameters such as acceleration or speed of the wheelchair, should be programmed in advance, since this type of controls act as mere switches. These controls usually present very low physical demands but high cognitive demands. Some examples of non-proportional controls are reinforced joysticks, which have a resistant design suitable for people who do not control their movements sufficiently or sensitive mini-joysticks, small size and highly sensitive joysticks. Designed for those users who cannot control a joystick, there are alternatives such as switch trays and systems with a single button. For those users who do have mobility in the head, it is also common to use proximity sensors arranged in a headrest. Finally, one of the most used options for people whose mobility is very limited is the use of a pneumatic system, which allows the control of the wheelchair through the spiff and spuff. Table 2 shows a collection of non-proportional control systems for wheelchairs that can be found on the market today.

Despite the wide variety of controls that are currently available on the market, the varied diversity of conditions that can affect wheelchair users—both physical and cognitive—makes that current control devices do not cover the needs of all of them. For this reason, the scientific community continues to focus on the investigation of new techniques that make it possible to adapt the use of motorized wheelchairs to as many people as possible. Within the current state of the art, some of the most relevant studies in this field focus on the following fields of application:

- Voice control: the great advance of the automatic speech recognition technologies during the last years has made this approach to be one of the most widespread method in human-machine interaction systems. There are examples of its application in the field of medicine [20], industry [21] or home automation [22]. In the same line, there are several works in which the automatic speech recognition technology has been used for the control of motorized wheelchairs. The most common type of control by voice is based on the detection of different keywords that act as commands to perform a particular movement. We can find examples of this type of systems in [23,24]. Another alternative to control a wheelchair by voice is presented in [25]. In this case, the control is based on the buzzing performed by the user. The control of the wheelchair is carried out based on the variations in the fundamental frequency of the user’s buzz, which is obtained through an accelerometer that measures the vibration of his vocal cords.
- Artificial vision: The use of artificial vision has been widely used in the field of assistive technologies, allowing to overcome certain limitations in different needs of users, such as mental functions, mobility problems, sensorial substitution or assistance services [26]. Within this field, it has also been applied as a wheelchair control system. Its most widespread use is based on the detection of the user’s eyes movement and sometimes it is also combined with the eye blinking. The combined use of both parameters is used as a control method for wheelchairs, as we can see in works like [27] or [28].
- Deictic control: in previous studies such as [29,30], it is proposed to use a deictic approach to control an electric wheelchair. Using laser devices and a webcam, these systems are able to perceive the environment. Thus, the system allows the user to indicate the destination to which he wishes to go and that the wheelchair moves to that point autonomously.
- Brain interface: Electroencephalography (EEG) is a technique that allows the recording of the bioelectric activity of the brain by means of electrodes applied on the scalp. The use of this technique, combined with the use of algorithms that classify different patterns in the recorded signal, has also been used as a possible control system [31,32].
- Electromyographic signals: analogous to the use of electroencephalographic techniques, electromyographic signals (those produced by the muscles during the contraction and relaxation processes) have also been studied as a suitable control system for wheelchairs. We find examples in Reference [33], where a combined use of this technique is made with artificial vision techniques to detect gestures made with the head; in Reference [34], where an electrooculogram system is used to measure the movement of the eyes; or in Reference [35], where electrooculogram and electromyography techniques are combined to recognize different gestures of the user’s head.
- Autonomous navigation systems: through the use of different situation awareness techniques, either through visual tracking [36], BLE beacons [37] or others, it has been possible to develop systems that allow wheelchairs to autonomously move in indoor spaces. Within these systems, there are two main trends: the control of wheelchairs in a 100% autonomous way, without the intervention of the user of the same, or the use of these techniques as driving assistance systems, such as for collision prevention [38] or semi-autonomous driving systems [39].

As it has been shown, due to the advance in the development of sensors and artificial intelligence techniques applied to the control of wheelchairs, the number of different control interfaces has significantly increased during last years. This fact makes that the traditional approaches of designing wheelchair interaction systems, conceptualized to be controlled by a single interface, is currently obsolete. Given this fact, it expected that, as an alternative, similar solutions to those adopted in the field of robotics will come to light, where during the last years, many proposals that encapsulate solutions to the most recurrent problems in this field have arisen: Robotic Software Frameworks (RSFs). These frameworks propose integral solutions to build general purpose robotic systems through a set of tools, libraries and algorithms. RSFs speed up the development and deployment of software and hardware components in a simple way [40]. Several examples of RSFs can be found in the literature, such as Player 2 [41], Open Robot Control Software (OROCOS) [42], Robotic Operating System (ROS) [43], OpenRTM [44] or OpenRDK [45]. The main utilities that provide this type of frameworks are focused on the following areas: middleware for distributed robotics, introspection and management tools, advanced development tools, robot interfaces and drivers, robotics algorithms and simulation and modelling of the system [40].

Within the field of robotics, the use of Multi Agent Systems (MAS) has also been widely applied to the definition and construction of robotic systems, giving rise to the research field of Multi Agent Robotic Systems (MARS) during the late 80s. Since then, MARS have been very popular for solving real-world problems that are better modelled by using a set of agents instead of a single agent [46], being favoured by the intrinsic characteristics of MAS—heterogeneity, modularity, scalability, parallelism and flexibility and robustness against faults. This set of characteristics make MAS especially appropriate for the construction of complex systems [47]. Two are the central areas in which MARS have been used: (i) multiple robot cooperative systems, where a set of robots works together to solve complex tasks, such as collaborative search and rescue [48], collective transportation [49], construction [50] or exploration and mapping [51] and (ii) multi-agent control systems, in which a group of agents cooperates to control a single robot in a distributed manner [52]. This approach is used mainly when the robot is composed of a large number of hardware and software components that require a modular architecture. In the same way as the RSFs, there are different platforms that allow creating and designing MAS in an agile way, such as JADE [53], MASON [54] or PANGEA [55].

After carrying out a study of the current state of the art regarding wheelchair control systems, it has been found that many different projects which new control alternatives for determined mobility problems are emerging. However, the integration of these control systems in a conventional motorized wheelchair model is not easy and it requires expensive processes at the software and hardware level. Therefore, the need for the development of a platform that will allow the control of any type of chair independently of the manufacturer, supporting the use of different devices and techniques for the control of motorized wheelchairs is detected. This work arises with this motivation, considering that the use of MAS, and more specifically VO, is the most suitable technology for this purpose, since it favours an efficient work and resources distribution and facilitates the control of the nodes where the agents are deployed. Furthermore, this work also covers the design and deployment of a set of alternative control interfaces that allow not only to verify the correct functioning of the developed platform but also to investigate in alternative control systems, so that in this way, the social inclusion of people with mobility problems is favoured.

## 3. Proposal

In this section, the proposed system is detailed. It consists of the construction of a hardware device that allows controlling any type of wheelchair regardless of the manufacturer. To validate the correct functioning of the system, different control techniques have been designed and implemented. Furthermore, the first functional prototype has been integrated into a conventional electric wheelchair (Quickie S-646 model). Due to the complexity of the proposal, it has been decided to divide it into different sections. Section 3.1 presents the design of the control hardware device and its integration with a conventional wheelchair, Section 3.2 presents MAS architecture that supports the operation of the wheelchair control system and in Section 3.3 the different implemented control interfaces are explained: through a mobile device (Section 3.3.1), by means of commands from voice (Section 3.3.2) or by an electroencephalography device (Section 3.3.3).

### 3.1. Hardware Control Device

Currently, the different existing alternatives for the control of wheelchairs are based on a single model or brand. For this reason, when the different institutions carry out new studies and designs of wheelchair control mechanisms, they are forced to carry out costly processes of adapting the control mechanism [56], resorting to the construction of prototypes that emulate wheelchairs or real situations [57,58], or even to make use of virtual simulators [59,60]. Under no circumstances (as for instance happens in the automobile sector with the CAN bus) efforts are being made to create a standard communication protocol that allows controlling all the wheelchairs on the market from different peripherals with a universal protocol.

Given this situation, the need to build a hardware device that allows to control any type of chair regardless the manufacturer has been raised. The main objective is that people with different disabilities can control by themselves any kind of wheelchair using different control interfaces in a simple manner. In this way, they will be able to perform movements that they would not be able to perform with conventional wheelchairs, gaining independence and allowing caregivers or family members to have a noticeable reduction of their workload. On the other hand, promoting the use of standards in the control of motorized wheelchairs, will make easier the work in the alternative control interfaces field of research. This will prevent the adaptation of existing wheelchairs or the construction of specific wheelchairs for the work to be developed by researchers. This will allow them to focus on the development of new technologies which aim to improve the social integration of disabled people in the field of mobility.

**Figure 1:**
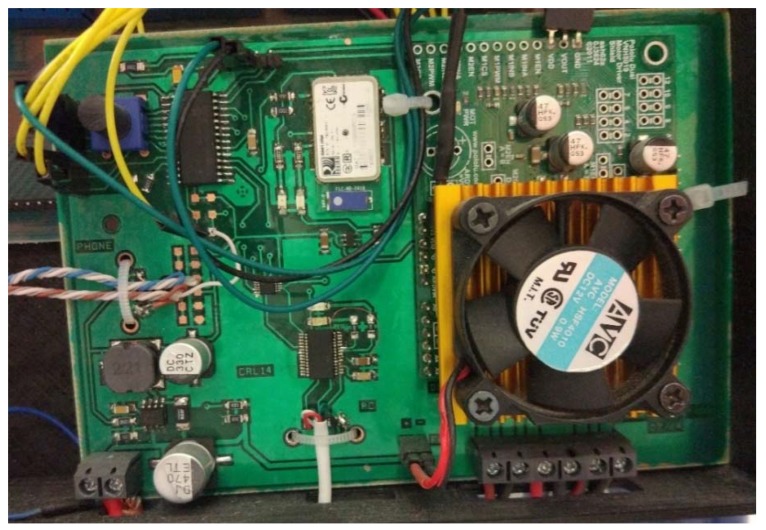
Printed circuit board.

As a central part of the system, responsible for the management of the wheelchair motors control, it has been necessary to develop a printed circuit board that allows communication between control devices (Figure 1). The main component of this board, which is in charge of the control of the system, is a Microchip PIC16F88 microcontroller. It is responsible, among other functions, for communicating the orders sent by the user through the control interfaces to the wheelchair motors. For this process, it has been necessary to incorporate drivers on the board to control the delivery of power to the motors. Pololu Dual VNH5019 drivers have been chosen for this purpose. To offer a wide alternative when choosing the control device for the wheelchair to be used, we have opted for the incorporation of two communication interfaces: a wired one, through a USB connection and a wireless one, via Bluetooth technology, which is supported by the use of a Microchip RN-41 chip. The main reason to incorporate a wireless communication method is to offer the possibility of controlling the wheelchair remotely. This functionality is specially designed to enable different control systems for situations in which the user of the wheelchair is assisted by another person, such as family members, nursing home staff, or airport personnel.

**Figure 2:**
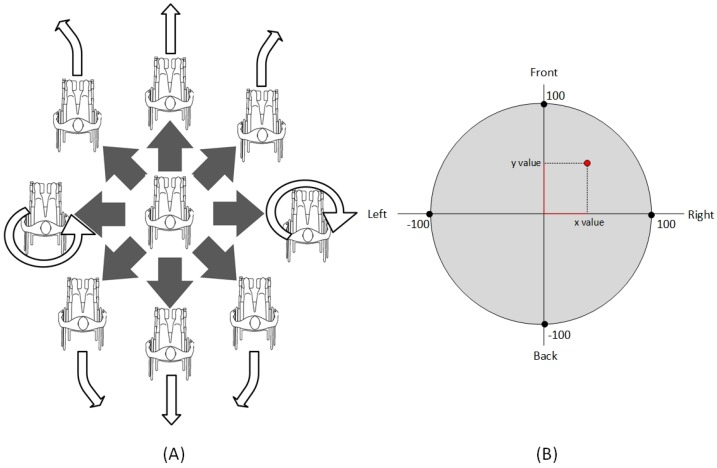
(**A**) Behaviour of the chair by non-proportional commands; (**B**) Range of values for chair control.

As it has been proven in the study of the current state of the art regarding wheelchair control devices, there are two types of control in the industry which are clearly differentiated and widely used: proportional controls and non-proportional controls. In order to be able to support both types of control, in the communication protocol between the developed board and the control devices, commands that allow the use of both alternatives have been incorporated. In this way, to perform a non-proportional control, a total of nine commands have been included, corresponding to the movements of: front, back, left, right, four diagonal movements and stop. Figure 2A shows the correspondence between the different non-proportional commands and what is the direction of movement that occurs in the chair. Since through this type of control it is not possible to regulate the speed of movement of the chair, an additional command which allows to configure the power applied by the motors has been included. It is therefore possible to apply five different power levels, being level 1 the one of lower power and 5 level with the highest power.

Regarding the proportional controls, we have been inspired by the operation of the most common proportional control: the joystick. In order to emulate its operation, the control devices will be able to send commands to the developed board whose content implies not only a directional component of the movement to be carried out but also relative to its speed. With that purpose, these commands are composed of two values that represent the displacement in the *X* and *Y* axes, for which the value 0.0 corresponds to the central position of the joystick (chair stopped). The admitted values for the axes are defined according to:

$$X^{2}+Y^{2}\leq 100^{2}$$

The positive values for the *Y* axis correspond to a forward movement and the negative values to a backward movement. In the case of the *X* axis, negative values correspond to a movement to the left and positive values to a movement to the right. On the other hand, the higher the absolute value of the sent command, the higher the motor power delivery is, reaching the maximum value with 100 (See Figure 2B).

In addition, a set of messages that inform the rest of the connected devices of the general state of the system have been included in the communication protocol. In this way, information regarding the battery charge status, the speed of the wheelchair, the temperature of the control board, or the status of additional components such as the lighting system or proximity sensors is provided. In the case of a traditional wheelchair, this type of information is not presented to the user, while it can be very useful. In the developed functional prototype, an interface for the visualization of this data has been implemented, as it will be shown later.

**Figure 3:**
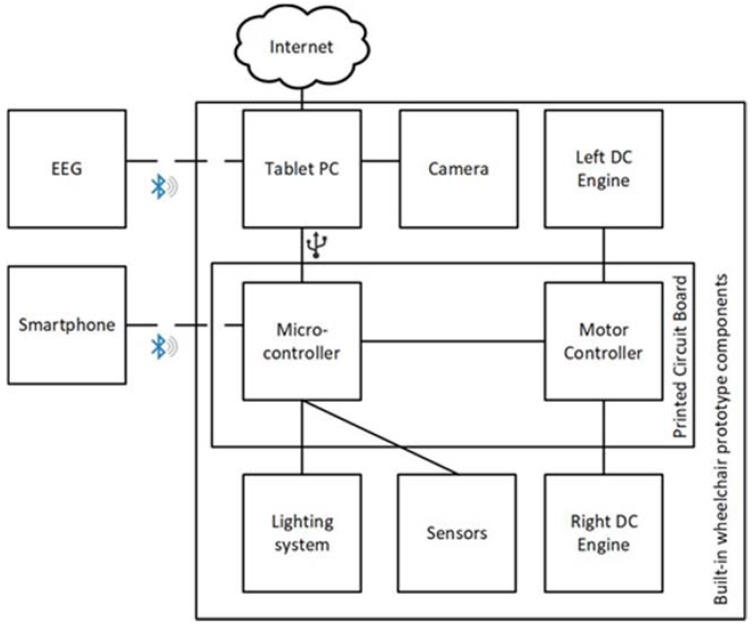
Prototype components.

As it has been previously introduced, in order to carry out a series of tests that validate the correct functioning of the proposed system (as carrying out new investigations on control interfaces for wheelchairs) a functional prototype where the proposed system is integrated has been developed. Figure 3 shows the main components of this prototype. As it can be seen, the central component of the system is the printed circuit board. It is designed to communicate with other devices in a bidirectional manner, so that it sends information about the current state of the system and receives commands with which to manage the operation of the wheelchair. In order to perform an effective control of the power applied by the motors, a specific controller has been incorporated for these, also integrated in the printed circuit board.

**Figure 4:**
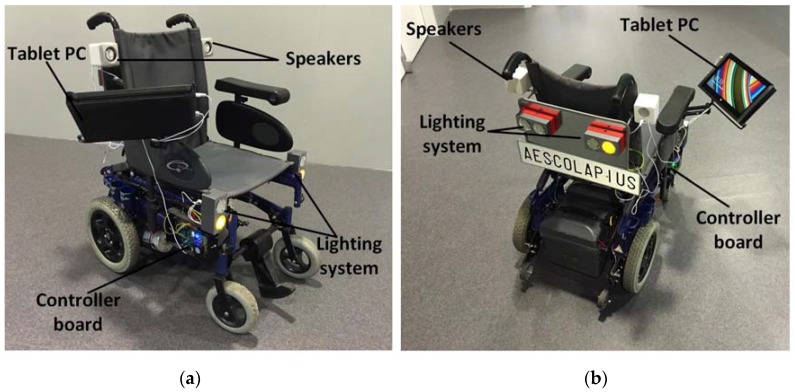
(**a**) Prototype front picture; (**b**) Prototype back picture.

To represent the information about the state of the chair (level of battery charge, speed, or temperature among others), an application has been developed that in this case runs on a Microsoft Surface tablet that has been incorporated into the prototype. Thanks to the use of this application, real-time viewing of images captured by a camera located at the back of the chair is also done, allowing users with mobility problems in the neck to see what happens behind them. This tablet is connected to the control board via USB, which allows it to send orders, as well as receiving information. To improve the autonomy of the tablet battery, a current inverter has been incorporated. It is connected to the wheelchair batteries and allows the tablet to be fed through them. In order to improve the safety of the user of the chair, a network of ultrasound sensors (HC-SR04 model) has been included in the prototype. It allows the detection of possible obstacles in the chair’s trajectory, which allows us to act accordingly by stopping the wheelchair to avoid a collision. Also related to the safety of the user, the prototype has been provided with a lighting system, which on the one hand improves the visibility of the user and on the other one, it helps to warn other pedestrians of the movements made by the user of the chair, since it acts as an indicator of certain movements, such as lateral or backward movements. Finally, three control interfaces have been developed for the wheelchair: (i) making use of the mobile phone, which is connected to the board designed through Bluetooth; (ii) by voice commands, processed in the application of the tablet; (iii) through the data collected by a wireless electroencephalography device, whose signals are processed and interpreted on the tablet. These control mechanisms are described in detail in Section 3.2. Next, two photographs of the front (Figure 4a) and back (Figure 4b) of the developed prototype are shown.

### 3.2. Multi-Agent Architecture

In the case of the study that has been carried out, the architecture that supports the operation of the wheelchair control system is based on the use of VO of agents. The main reason to follow this kind of methodology is to provide the system with the inherent advantages of the use of VO, which are aimed at achieving more open and dynamic systems, in which agents have a set of roles and standards that determine their behaviour. The possibilities offered by the organizational aspects of this type of system can largely determine the flexibility, dynamism and openness of the multi-agent system. As a basis for the implementation of the multi-agent system, PANGEA has been chosen [55]. It is a multi-platform agent platform created by the BISITE research group of the University of Salamanca for the development of open multi-agent systems, especially those that include organizational aspects. The platform allows the integration of organizations and offers a set of useful characteristics. From the agents’ perspective, different models of agents are included, such as BDI and CBR-BDL, while from an organizational perspective, it can be easily managed. Any organizational topology is allowed within this platform, while a business rules engine ensures the compliance with the standards established for the proper operation of the organization. Furthermore, a set of services is included which allow to dynamically reorganize the agents’ organizations or distributing tasks and balancing the workload. Furthermore, a useful set of tools is included for end users, as graphic tools to control the agents’ life cycle, debugging and service discovering tools or an interface to oversee the organizations. From a communicational perspective, PANGEA allows the use of different communication protocols. Some of these protocols are focused on communication between embedded devices, so it is a platform that matches the requirements necessary for the development of this system. Moreover, the possibility to interact with FIPA-ACL agents is supported.

**Figure 5:**
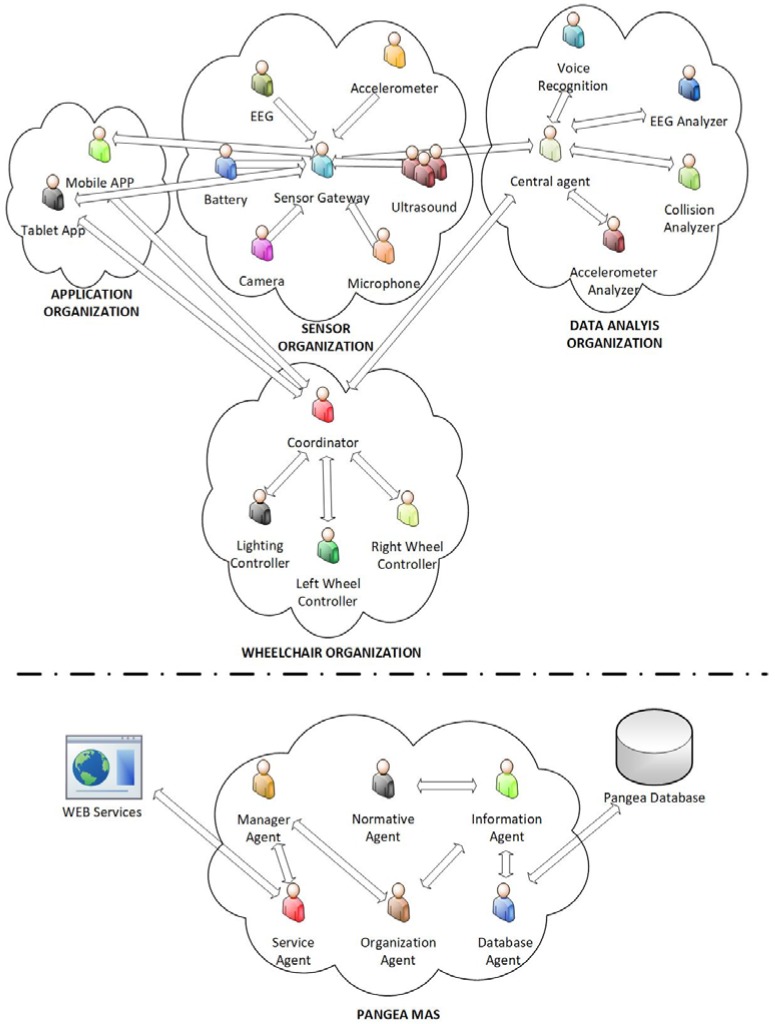
Multi-agent Architecture.

Figure 5 shows the organizational structure of the proposed multi-agent system, responsible for the management of the platform. On the one hand, the upper part is specifically designed for this case study, while the lower part shows those agents proper of PANGEA. Next, the functionality provided by the different organizations of agents that make up the system is detailed:

- Sensor organization: the purpose of this organization is to collect data from a set of hardware sensors for subsequent representation and analysis by other agents in the MAS. As a central part of this organization, the Sensor Gateway Agent is responsible for collecting the data of the agents embedded in the sensors present in the system, normalizing the collected information and transmitting it to those agents of the rest of the organizations that request it. In the case of study presented in the article, the sensors involved in this organization are: battery (knowing the battery level of charge), EEG, accelerometer (obtaining the smartphone accelerometer data), ultrasound (proximity sensor), microphone and camera.
- Data analysis organization: based on the data provided by the sensor organization, the agents that are part of this organization are in charge of analysis to carry out decision-making processes related to the control of the wheelchair. Within this organization, a central agent is incorporated. It is in charge of sending the necessary data from the sensors to the rest of the agents of the organization, who are responsible for analysing it. After these processes, the central agent will receive back instructions regarding the control of the wheelchair. This agent will communicate these instructions to the organization in charge of managing the wheelchair organization. In the event that the decisions made by the agents of this organization come into conflict, the central agent is responsible for managing their priority. Among the other agents of the organization are: the voice recognition agent. which, through the audio signal collected by a microphone, uses a speech recognition system to interpret different commands with which to control the chair; the EEG analyser that analyses the data collected by an electroencephalogram device to control the wheelchair; the Collision Analyser, which attending on the signals collected by the proximity sensors, is responsible for taking the control of the wheelchair to avoid possible collisions and the Accelerometer Analyser, which analyses the data obtained from the smartphone embedded accelerometer in order to control de wheelchair and also to detect possible falls of the smartphone.
- Wheelchair organization: responsible for carrying out the control on different components of the wheelchair. The Coordinator Agent is the central agent in charge of the coordination of the organization. The Right and Left Wheel Controller agents are in charge of the individual control of each of the motors and the Lighting Controller Agent is responsible for the control of the lighting system that incorporates the wheelchair.
- Application organization: For the end user to be able to view the information collected from different sensors, a tablet software application has been developed. The agent responsible for collecting this information and also responsible for the visualization layer of the application is the Tablet App agent. This organization also includes the Mobile App agent, responsible for managing the wheelchair control system based on the smartphone touchscreen.

On the other hand, the proposed architecture is based on the use of the agents provided by the PANGEA platform [55]. These agents are responsible for tasks such as: (i) supervision of the correct performance of tasks (manager agent); (ii) the registration of the agents and services present in the system (information agent); (iii) control of the correct operation of the services offered by the agents and their distribution through web services (service agent); (iv) guarantee the correct compliance of the rules defined in the organizations (normative agent); (v) access to database (database agent); or (vi) management of the different organizations that make up the system (organization agent).

**Figure 6:**
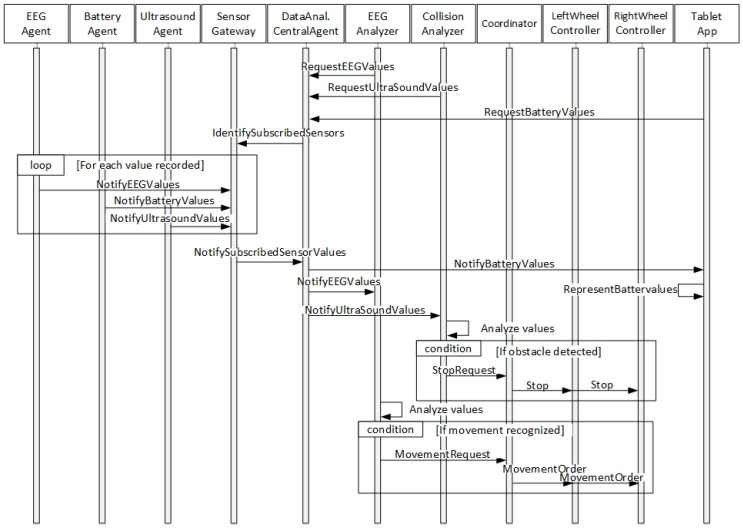
Collaboration-sequence electroencephalogram (EEG) control interface diagram.

The general operation of the processes occurring in the platform for a specific control interface can be seen in Figure 6. In this case, EEG control interface has been chosen to represent the platform’s operation processes. Firstly, agents within Data analysis organization that are involved in the selected control interface perform a subscription request to the central agent of this organization. In this request, these agents specify which data coming from the hardware sensors is needed for their proper functioning. In this case, two agents will work in parallel: EEG Analyser and Collision analyser. EEG analyser is in charge of performing the portable EEG device data analysis to infer which movements does the user want to perform, while the Collision analyser works with the ultrasound sensors data to detect possible environment collisions. Thus, the data subscription request performed by these agents are the EEG and the ultrasound sensors data in each case. Subsequently, the data request from this organization to the sensors organization is done to the sensor gateway agent, which collects all the data coming from the different agents in charge of obtaining information from the different deployed sensors. As EEG and Ultrasound agents send their corresponding hardware data to the sensor gateway, this information is redistributed to the data analysis central agent (only subscribed information is sent). As the requested information is provided to each analyser agent, it is evaluated by each of the agents. In the case of the EEG analyser, if a certain pattern associated with a movement is recognized, a movement request is performed to the wheelchair organization’s coordinator agent, while if the Collision analyser detects a possible impact, a stop request is done. When a request is done to the coordinator agent, it is in charge of evaluating it according to its priority. For instance, in this case, the collision analyser requests will always have a higher priority. Once the requests are accepted, the corresponding order is sent to the wheel controller agents to perform the physical movement. Additionally, Figure 6 also shows how the information represented in the tablet App is provided through the agents’ organizations. The battery information flow is provided as an example.

### 3.3. Control Interfaces

#### 3.3.1. Smartphone

In some cases, users of wheelchairs (manual or electric) cannot drive independently due to limitations in the environment (large slopes, reduced space), or physical limitations. In these situations, it is necessary to resort to a second person who assists the user of the wheelchair and does not always have the necessary strength to move the chair without having to make a great effort. Obviously, controlling a motorized wheelchair while walking by using the traditional joystick is not easy. For this reason, to facilitate its operation by the attendees, certain models of electric wheelchairs have controls which are specially designed for attendees. These controls are usually located in the back of the wheelchair to ease its use. In this way, it is the assistant who controls the direction of the chair, while the motors make the effort to move the chair. Although this option is available for several models of chairs at present, not all models offer the possibility of integrating a system of these characteristics and in case of doing it, the price is generally high.

Faced with this situation, it has raised the possibility of developing a control system for assistants that is affordable, accessible to anyone and intuitive to use. To carry out this system, we have chosen to use smartphones. This decision is fundamentally motivated because its penetration of use is increasing, so that a large part of the population has one (reaching a penetration of 78% of the total population in the case of the US, or 87% in the case of Spain [61]) and, in addition, they have great connectivity and ways of interacting with them. In this paper, we propose three alternatives for controlling a wheelchair through the mobile phone: one by using the accelerometer of the mobile phone and two by using the touch screen of the mobile phone. All these alternatives make use of the Bluetooth communication between the mobile device and the designed control board, sending through this communication channel the necessary commands for the movements of the relevant wheelchair.

**Figure 7:**
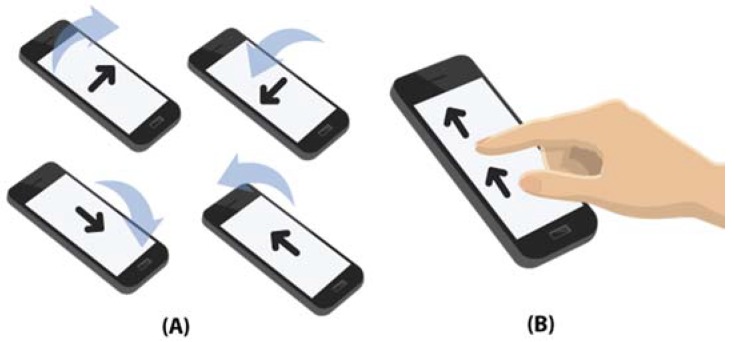
(**A**) Telephone inclinations allowed by the mobile device; (**B**) Interaction by touch control.

Most smartphones have an acceleration sensor embedded inside. This sensor allows to measure the inclination of the device in relation the ground. Using this sensor, a non-proportional control method for the wheelchair based on the inclination of the mobile phone has been developed. To do this, a finite state machine is defined with possible states (front, back, left, right and stopped). The transition between states will be made based on a series of thresholds for the values recorded by the accelerometer for the *X*, *Y* and *Z* axes. Figure 7A shows how the mobile phone should be tilted to make a movement in the wheelchair. For example, tilting the phone as shown in the upper left of the image corresponds to a forward movement.

Although the accelerometer-based control is very intuitive, the most common form of interaction with a smartphone is through a touch screen. For that reason, there may be users who are more comfortable using this element as a wheelchair control interface. On the other hand, it can be a more suitable control method for wheelchairs users that have good mobility in their hands but not enough strength to be able to easily use a joystick. This kind of control is a possible substitute for low cost control interfaces based on the use of touch panels, the price of which is generally high, such as the Switch-It TouchDrive 2 [18], which exceeds 3000€. To integrate this option in a conventional wheelchair, it will be enough to incorporate a small support for the mobile phone in the armrest of the chair, so that it is placed under the user’s hand. Two alternative touch screen-based control methods are proposed.

**Figure 8:**
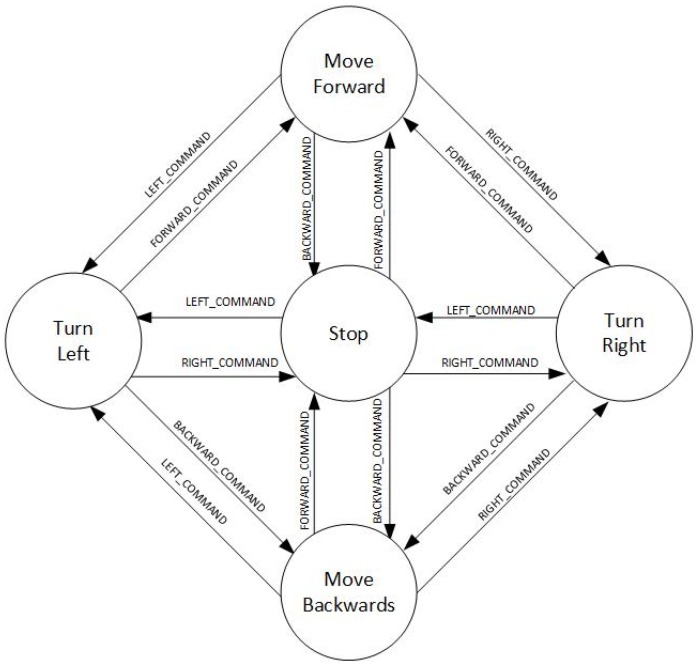
Possible states transition of the non-proportional touch screen-based control.

The first one is a non-proportional method, which allows the transition between 5 states (the same ones used in the case of the accelerometer). Figure 7B shows how to interact with the touch screen of the mobile device: sliding a finger in the specific direction in which the user wants to move. For example, to make the wheelchair to move forward, you must slide your finger from the bottom of the screen to the top. To cancel the movement, that is, to make the chair stop, the opposite movement has to be made, in this case from the top to the bottom. It will be possible the direct transition between all the movements, excepting the opposite movements (since they imply that the chair stops). Figure 8 shows the possible states transition of the non-proportional touch screen-based control.

Algorithms 1 and 2 shows the pseudocode of how the non-proportional touch screen-based control is implemented. As it can be observed, Algorithm 1 oversees analysing the press and release coordinates on the touch screen, while Algorithm 2 evaluates the results of Algorithm 1 to perform the corresponding movement requests: moving forward, backward, left, right or stopping the wheelchair.

The second touch screen-based method is a proportional control method that emulates the traditional joystick of a wheelchair. Although the kind of control offered by this method is analogous to the one offered by a joystick, its use is proposed for those people with partial mobility in their hand but who can move a finger in a controlled manner in a small space, in the same line as other control devices based on the direction of the finger, such as the DX-RJM-VIC [62].

**Algorithm 1.** onTouchEvent.
**Input:**
*motionEvent*

**Output:**
*None*
**Variables:***xi, yi*: point when press, *xf, yf*: point when release; *xr, yr*: relative coordinates
**if**
*event.getAction == MotionEvent.ACTION_DOWN*
**then**
  *xi = event.getX()*  *yi = event.getY()*  **else if**
*event.getAction() == MotionEvent.ACTION_UP*
**then**    *xf = event.getX()*    *yf = event.getY()*    *yr= yf – yi*    *xr = xf – xi*    **if**
*abs(xr) >= abs(yr)*
**then**      **if**
*xr > 0*
**then**        **if**
*a < 1*
**then**          *a++*        **else if**
*a == 1 || a == .1*
**then**          *b=0*        **end if**        *sendCommand(a,b)*      **else**        **if**
*yr > 0*
**then**          **if**
*b < 1*
**then**            *b++*          **else if**
*b == 1 || b == −1*
**then**            *a = 0*          **end if**          *sendCommand(a,b)*        **else**          **if**
*b >= 0*
**then**            *b++*          **else if**
*b == 1 || b == -1*
**then**            *a = 0*          **end if**          *sendCommand(a,b)*        **end if**      **end if**    **end if**  **end if**

**Algorithm 2.** sendCommand.
**Input:**
*a,b*

**Output:**
*None*

**if**
*a==0 && b == 0*
**then**
  *requestStop()*
**else**
  **if**
*a == -1*
**then**    *requestLeftMovement()*  **end if**  **if**
*a == 1*
**then**    *requestRightMovement()*  **end if**  **if** a == 1 **then**    *requestForward()*  **end if**  **if**
*a== 1*
**then**    *requestBackward*  **end if**
**end if**


#### 3.3.2. Voice Control

In order to make the use of a motorized wheelchair more accessible to those people who cannot perform physical movements, an additional control interface based on voice control has been developed. By using different voice commands, the user can control wheelchair movements in a non-proportional way. Despite being a non-proportional control, it also incorporates a command that allows controlling the speed of the chair at different levels. On the other hand, by incorporating this type of interface, the possibilities of interaction with the user multiply. To take advantage of this potential, additional functionalities have been incorporated, so that users can check the news or weather information, switch on and off the lights we have incorporated into the system or send an emergency signal.

The application that handles voice recognition management runs on the Microsoft Surface tablet and is active by default. To perform speech recognition, the voice recognition API of the .NET Framework has been used. The main advantage that this API offers over others is that it performs the voice recognition process locally, without the need to interact with any external server. In addition, it allows to establish grammatical restrictions to a finite set of alternatives, which in this case correspond to the commands admitted by the system. The use of technologies that depend on a stable network connection could compromise the user’s security when it is unstable. To prevent the commands that control the movement of the wheelchair from being activated unintentionally, since they could appear in any normal conversation that the user kept, a keyword has been included in all the commands, except for the command that makes that the wheelchair stops. Next, Table 3 collects the commands admitted by the system together with the system response for each of them.

#### 3.3.3. EEG

Many wheelchair users suffer from diseases that prevent them from using the motion interfaces presented in the previous sections. For example, diseases such as amiatrophic lateral sclerosis (ALS) or spinal cord injuries that are likely to result in total or partial paralysis. It is therefore essential to include a motion interface that allows wheelchairs to be used for this type of person. This is where the use of EEG-based technologies is proposed as an alternative to the methods proposed above.

**Figure 9:**
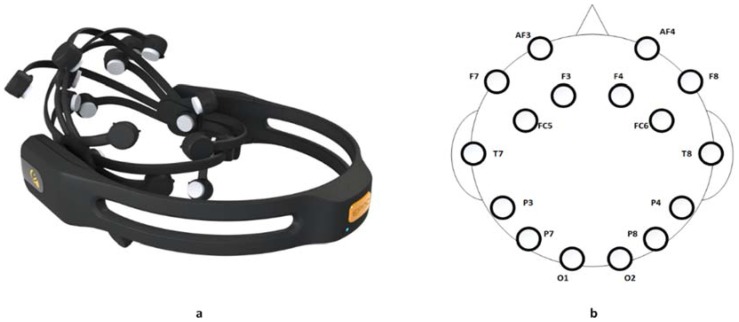
Electrocardiogram (ECG) interface. (**a**) EPOC+ helmet of Emotiv; (**b**) Distribution of EPOC+ helmet sensors in the head of subjects.

The EPOC+ helmet designed by the Emotiv Company (San Francisco, CA, USA) is used to implement an intelligent system capable of using EEG-based technology. It is a Neuroheadset device. An image of the device used is shown in Figure 9a. The helmet is made up of a total of sixteen sensors that work with a conductive liquid of the current, such as contact lens liquid. Fourteen (AF3, AF4, F7, F8, F3, F4, FC5, FC6, T7, T8, P7, P8, O1, O2) of these sensors are data channels and the other two (P3, P4) are the so-called location sensors. Figure 9b shows the distribution of the different sensors once a person has placed the helmet. As it can be observed in Figure 3, the wireless electroencephalography device is directly connected to the Tablet PC and not to the platform built-in Bluetooth. The reason is that it requires to be connected to a specific USB Bluetooth receiver developed by Emotiv.

The Emotiv EPOC+ helmet is able to recognize affective, expressive and cognitive cases. To do this, it measures brain activity by means of an electroencephalogram. Cognitive casuistry is what will be taken into account to implement the system that will allow the wheelchair to move. The system is designed in such a way that the user can use abstract thoughts or observe specific images to guide the wheelchair.

In this way, data will be collected from various people to test the effectiveness of the system and the designed motion interface. Each of these people will be made to think of a series of abstract concepts, specifically four, in such a way that each of these thoughts is later related to the four movements that can perform the electric chair, left, right, front and back. In the same way, four arrows will be related to each of the four previous directions. In the latter case, the user will have to react to the stimulus by looking at each of the four arrows.

For each of the users, different datasets will be extracted since each one of them thinks in different concepts and they do not have the same reaction when visualizing the images. This data set will be obtained each time individuals use the interface. With each use, there will be a small initial training stage of about 40 s. This stage of the user’s initial adaptation to the helmet is necessary since, although the same thoughts are always related to each of the thoughts, the brain response may not be the same in each of the interface applications. Once this training phase is completed, the generated dataset will be used to train the model that will then be used to move the wheelchair. In order to obtain these models, it is necessary to apply a pre-processing that helps the identification of movement patterns. The Results and Conclusions section will show how several models with different machine learning techniques are constructed in order to determine which of them offers the best results when evaluated. The algorithms to be used are tree-based methods and decision rules, role-based learning, Bayes’ theorem, as well as meta-classifiers. All of them will be explained later in this section.

##### Processing of Raw Data

The different steps of the processing of raw data obtained from helmet readings are explained below.

##### Signal Sampling

As indicated in Section 3.3.3, the Emotiv EPOC+ helmet is capable of obtaining 14 bioelectric records from its fourteen sensors. Each of these signals is sampled at a frequency of 128 Hz, that is, for every second 128 samples per sensor are obtained. In this way, the time domain signal is obtained for each sensor. However, it is necessary to pass the signal to the frequency domain in order to be able to better recognize the patterns within each signal, just as it is performed in work [46].

From each signal, a 2-s window has been chosen for the Fourier transform, with a shift of 0.5 s. In other words, 256 values are selected, with a shift of 64 values. For each time window, the Fourier transform is applied. Unlike other works that recommend the use of windows of 5 s, in this work has been chosen 2 s as window by the delay that represents when detecting the movement and perform it.

##### Obtaining the Fourier Transformation

The Fourier Transformation is a mathematical transformation to pass a signal from the time domain to the frequency domain. Given a signal in the time domain s(n), the fragmented signal in I windows is denoted as $s_{i}\left(n\right)$, where i∈I denotes the window being transformed. For each window, the Discrete Fourier Transform (DFT) is calculated as shows in [46]. As the wrap function, the Hanning window has been chosen which, in the interval n ∈ [0, N−1], is the formula shown below.

$$w\left(n\right)=0.5\left[1-\cos\left(\frac{2\pi n}{N-1}\right)\right]$$

where N is the number of examples selected, in our case there are 256, as explained above in the signal sampling description.

##### Transformation of Data into Decibels

The transformation of the DFT into decibels allows the logarithmic nature of the decibel to simplify operations with low power values. This is the case of Emotiv EPOC+, where the records of the signals are given in μV. Formally, the transformation of the DFT from an S(k) signal into decibels is as follows:

$$S_{dB}\left(k\right)=\;20*\;\log_{10}S\left(k\right),\;1\leq k\leq K$$

**Figure 10:**
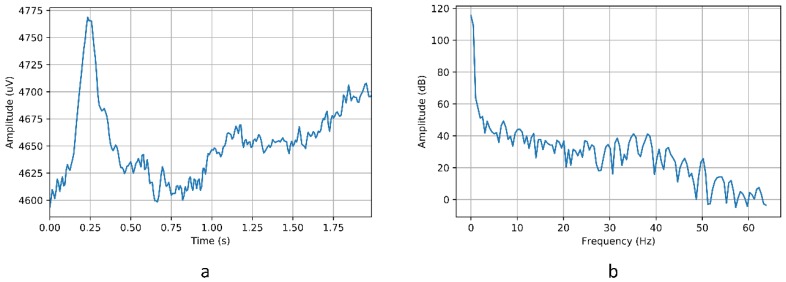
Signal corresponding to the “left” movement of the AF4 sensor. (**a**) Signal in the time domain; (**b**) Signal in the frequency domain.

Figure 10 shows the transformation of data in the time domain into the frequency domain. The images show the signal corresponding to the “left” movement recorded by the AF4 sensor. Once the data has been obtained in the frequency domain, the model is constructed from which the movements to be carried out by the chair will be detected.

It is important to mention that the extraction of data from the encephalogram is obtained by way of the EmotivPRO software (Version 1.2, San Francisco, CA, USA), also provided by the company Emotiv under subscription. Once the data has been obtained, they must be pre-processed in order to pass the data found in the time domain to the frequency domain by applying Fourier transforming.

##### Model Construction Techniques

As for the algorithms that will be used to determine the movement of the chair, according to the data pre-processing, are explained below. With these techniques, we will analyse which of them is best suited to the casuistry of the problem, in order to make a classification as accurate as possible.

##### RIPPER

The Repeated Incremental Pruning Produce Error Reduction algorithm or better known by its acronym RIPPER is an algorithm that evolves from the IREP (Incremental Reduced Error Pruning) algorithm and in turn this last algorithm is a combination of the REP (Reduced Error Pruning) algorithm and the divide and beat technique.

The IREP algorithm was described by Fürnkranz and Widmer [63]. It is a technique that makes use of decision trees to determine the class to which the different instances correspond. Cohen improves this algorithm and called it RIPPER. The improvements of this algorithm include the pruning function of the tree, a new criterion for stop addition process based on heuristics and a new optimization step after the rule set. For more information about the implementation of the RIPPER algorithm see the original article of Cohen [64].

##### C4.5

Algorithm C4.5 is an evolution of the original ID3 whose main advantage is that it allows numeric attributes to be incorporated into the logical operations carried out in the test nodes. Currently, there are new versions of this algorithm such as C5.0 but it is a commercial version. J48 is the implementation of C4.5 in Java and is available in data mining tools like Weka. It is one of the most used techniques together with CART since both allow the use of numerical attributes. C4.5 tries to minimize the width of the decision tree through heavy search strategies. To do this, two terms are defined, the gain and the rate of gain based on the information H(x) contained in a node x. Using only the criterion of gain, attributes with many values are favoured since they favour the division of elements into numerous subsets, to avoid this the concept of the rate of gain is added. The idea was proposed by Quinlan [65]. In this book, the researcher explains how the algorithm works.

##### Random Forest

The Random Forest algorithm is somewhat inspired by Bagging algorithms and the bootstrapping technique. The original idea of the Random Forest is to improve the reduction of variance in Bagging, reducing the correlation between trees that are generated without reducing too much variance. This idea was proposed by Breiman [66]. This is achieved during the stage of tree creation and growth, thanks to the selection of random variables that will compose the tree.

The algorithm does this by creating a series of random trees, where the random factor is determined by the variables that are selected to form the tree. In [66] the process is explained.

##### Bayesian Network

The Bayesian network-based algorithm is a probability-based model that relates to a set of random variables using an acyclic-directed graph that allows Bayesian inference to be used to determine the probabilities of unknown variables from others that are known. Bayesian networks are composed of nodes, one for each random variable and by directed arcs that relate the nodes. In addition, it is known that the probability of each node is conditional on that of the parent node. Full explanation about this algorithm is defined by Su and Zhang [67].

It is important to point out that in case any attribute has continuous values, it is necessary to make a discretization of the data, since if not this type of variables makes it very difficult to calculate the probabilities.

##### SVM

The SVM (Support Vector Machine) algorithm is one of the algorithms that will be applied for the classification task. The technique of this algorithm consists of constructing hyperplanes in the space in which the data are represented, so that these hyperplanes maximize the distances between the different classes. This technique is explained in more detail in the article written by Vapnik [68]. This technique is one of the most widely used classification techniques that use data in the frequency domain. In addition, this technique also offers very good results compared to other techniques.

##### Meta-Classifiers

The bagging algorithm comes from the words bootstrap aggregating. The technique consists of obtaining different training sets from an original one using the bootstrapping technique. Each of these datasets will be the input of a different classifier and with which each of these classifiers, which are usually different from each other, will produce a classifier model that is able to make decisions. Once these models have been obtained, each time a new instance is to be classified, it will be classified by each one of them. Each of these models can arrive at a different result in such a way that the multi-classifier has to say the class that will be offered as a result of the process by the majority voting technique. This technique was originally proposed by Breiman [69]. The boosting technique, on the other hand, uses a single classifier to generate the different models. These models are trained by different subsets of data that are also obtained through bootstrapping. In the algorithms that implement this technique, such as AdaBoost, each of the instances of the training subsets of each classifier receives an initial weight. Each of these subsets is trained during a given number of iterations. In each of these iterations the model error is checked and if it does not meet the satisfaction conditions, a new iteration is performed but updating the weights of the misclassified instances. In this way, each of the models that the multi-classifier would consist of would be trained. This will determine the resulting class by means of the class with greater weight than those returned by each of the internal classifiers of the multi-classifier. The idea was proposed by Schapire and Freund [70].

## 4. Results

This section evaluates the results obtained from the system proposed in Section 3. Firstly, we analyse the efficiency of the different algorithms proposed in Section 3.3.3 EEG. Once the results obtained by each of the algorithms have been presented, it will be necessary to determine which one best suits the characteristics of the system. In this section, we will first analyse the algorithms that will be responsible for discretizing between abstract thoughts and then analyse the algorithms that evaluate the data sets formed when users observe the images.

When determining the operation of the control interface formed by the EEG technology when the user thinks of abstract concepts, which are later related to the forward, backward, left and right movements, this operation is evaluated with the algorithms RIPPER, C4.5, RandomForest, Bayesian networks, SVM, Bagging with the RandomForest algorithm and Boosting also with the RandomForest algorithm. The versions of these algorithms are those implemented in the Weka tool [71].

Each of these algorithms will receive a total of five different datasets, one for each of the users with whom the tests have been performed, in order to determine their effectiveness. The five individuals selected are males aged 22 to 31. The datasets are obtained by the process described in Section 3.3.3. This data is used to evaluate the performance of the algorithms by generating the model and validating it with the 10-fold cross validation technique. The results of these tests are shown in Table 4.

**Figure 11:**
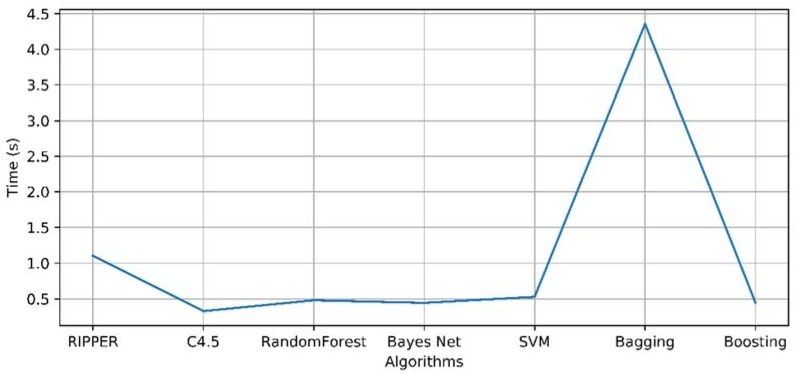
Evolution of model formation time.

As it can be seen in the image, all the algorithms obtain a very high efficiency, in all cases higher than 97% by validating each of their models with the 10-fold cross validation technique and a kappa also close to 1. These values indicate that each model offers a perfect performance for each of the individuals with whom the system has been tested. Therefore, it is essential that in this case we look at the time it takes to generate each model. This is best seen in Figure 11.

The above figure shows the average times of each of the models generated for each user. Looking at this graph it can be concluded that the best algorithm for dealing with the problem of abstract thought treatment is C4.5.

If we take into account when the efficiency of the system at the time of discretization, when the user visualizes the images of the arrows that are related to each of the four movements, it is evaluated with the same algorithms as before, RIPPER, C4.5, RandomForest, Bayesian networks, SVM, Bagging with the RandomForest algorithm and Boosting also with the RandomForest algorithm, also from the Weka [70] application.

In the same way as before, each of the algorithms is evaluated with the datasets of the five users and the 10-fold cross validation technique. The results of these validation tests can be seen in Table 5.

**Figure 12:**
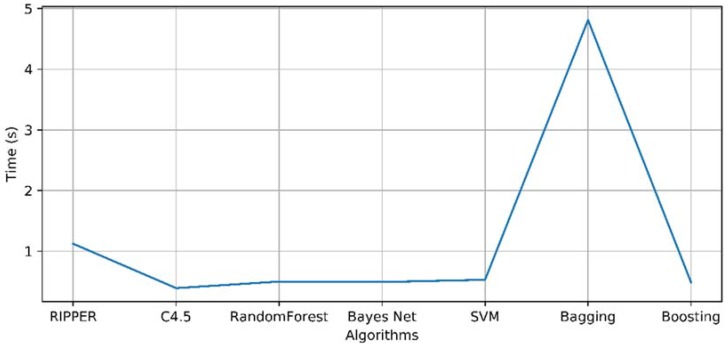
Evolution of model formation time.

Again, the models offer excellent results, both in terms of accuracy and kappa values. Therefore, it is again necessary to compare the time values in which it takes to generate each of the models. This can be seen in Figure 12.

Looking at the image, it is again the C4.5 algorithm that consumes the least time to generate the model. Therefore, this algorithm is selected from the rest of them to conform the model that will decide which image the user is viewing through the data received from their brain activity.

**Figure 13:**
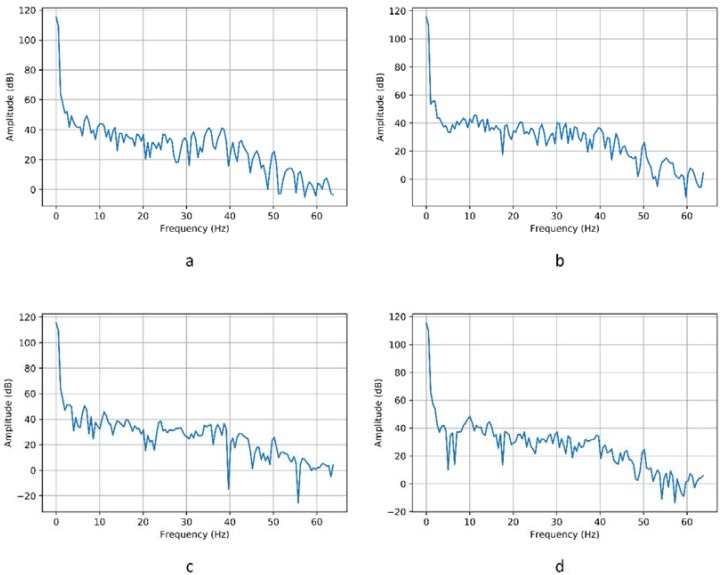
Signal picked up by the AF4 sensor of subject 1 in the first 2 s (**a**) Movement “left”; (**b**) Movement “right”; (**c**) Movement “forward”; (**d**) Movement “back”.

The high performance of all the proposed models is mainly due to data pre-processing. In which the time domain data is transformed into the frequency domain. We achieved this by applying Fourier transformation as well as with the enveloping function, Hanning, as explained in the subsection EEG. With this process, we achieve the models differentiating between each of the movements. Just as you can with the naked eye in Figure 13.

**Figure 14:**
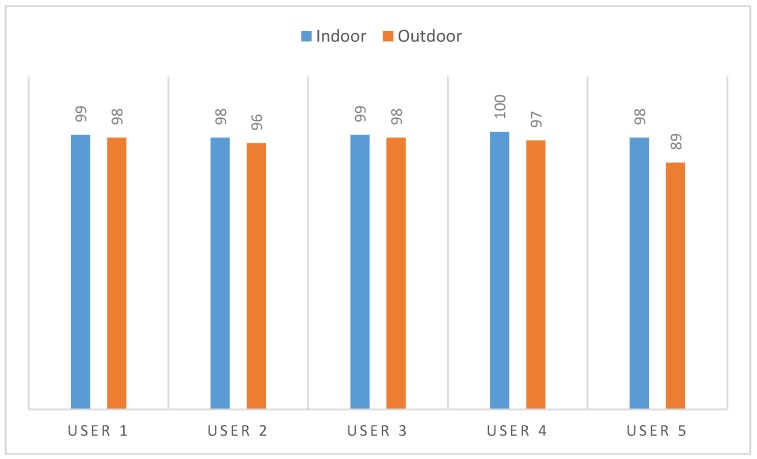
Accuracy of the speech recognition system.

Regarding the evaluation of the voice control interface presented in Section 3.3.2, a set of tests were carried out with five different users. It is important to remark that none of these users presented speech or pronunciation problems. In order to test the accuracy of the speech recognition system, five users participated in different tests. These tests were carried without using the wheelchair, since the scope of this tests were just to evaluate the performance of the speech recognition system, not its usability as a wheelchair control interface. During these tests, each user repeated 10 times each of the commands (see Table 3) in two different scenarios: an indoor environment and an outdoor environment (a relatively busy street). The correct recognition of commands by the system is of vital importance. Failure to recognize a command would mean that the wheelchair would continue to perform the same action it was performing. A more undesirable situation would arise when the system would mistakenly recognize a different command from the one that has been said. Figure 14 shows the percentage of correctly recognized voice commands during the tests in the indoor and the outdoor locations. Most of the errors produced in the systems were related to the non-identification of any of the commands. Only in 12 out of the 1000 spoken commands, the system confused one command with another.

**Figure 15:**
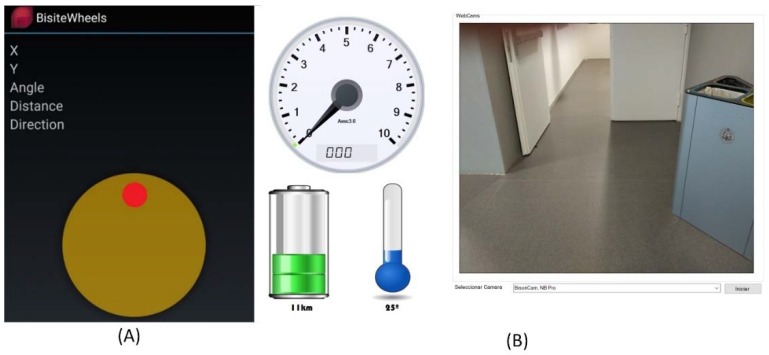
(**A**) Smartphone app interface; (**B**) Tablet app interface.

Figure 14 shows screenshots of the smartphone and the tablet applications. Figure 15A shows the interface of the mobile application that allows to control the wheelchair through the touchscreen. In order to control the wheelchair, the user has to slide a finger through the circle shown in the image, in the direction in which the user wants the chair to move. To stop its movement, user just has to stop touching the circle. Figure 15B shows the interface of the Microsoft surface tablet that is placed at the front part of the wheelchair which is used to present useful information to the user. As it can be seen in the figure, information regarding speed and electronic components temperature is represented. Furthermore, images taken from the web-camera located at the back of the wheelchair are shown in order to let the user have a broader vision of the environment. By doing this, the application allows users with mobility problems in the neck to see what happens behind them.

Since the precision of the smartphone control systems is not evaluable as it has previously done with the EEG and the voice controls, in order to evaluate them, some tests have been done with the purpose of obtaining feedback from the users regarding the accuracy and ease of use of these control systems. Tests were carried out on all the different control systems presented in Section 3 using the prototype wheelchair. Additionally, the use of the control interfaces based on the smartphone was also evaluated for its use by accompanying persons, not by users of the wheelchair. Five users carried out these tests, testing the control systems both indoors and outdoors following the same route. Finally, users assessed the accuracy and ease of use of the control systems using a numerical rating from 0 to 10. Results are presented in Table 6. Some conclusions were extracted from these evaluations and the users feedback: As it can be seen in the results, the two most accurate control methods are those based in the accelerometer and the touchscreen of the mobile phone. Users were very satisfied with the performance and ease of use of both control systems and all of them stood out the control based on the accelerometer for being more intuitive, which made this method of control the most comfortable to use while walking by the wheelchair. Regarding the control based on voice commands, users highlighted its ease of use, although they pointed out that they found it difficult to make precise turns due to the time required to speak the command to be made. Considering the EEG control system, it was the one which turned out to be the least easy to use. The main reason is that this system requires a more important process of adaptation and training by the user. Regarding the accuracy of this system as a control method, it proved to be the least efficient since it requires of a greater degree of concentration on the part of the user; it turned out to be an efficient indoor system but its use is more complicated in external environments, where plenty of external stimulus can affect in the user’s concentration.

## 5. Conclusions

The main conclusions obtained after the successful attainment of the objectives of this work are described below.

In this work, a platform based on virtual organizations of agents has been designed and developed to control an electric wheelchair from different external devices. The use of an open source MAS as PANGEA, has allowed to benefit from the advantages of this paradigm for the analysis, design and implementation of complex systems where the data sources and data processing are distributed. PANGEA provides all the required infrastructure to easily design and deploy the MAS, gathering and processing data and communicating with the users. It also enhances the possibility of designing different data access forms which allow a non-centralized information processing, enhancing the scalability faculties of the system. The use of a MAS architecture based on PANGEA, has allowed to develop the case study allowing the interconnection of agents developed in different programming languages: Java for the smartphone application, C # for the tablet application and C in the wheelchair controller. PANGEA is characterized for being an architecture that allows the connection of different devices in a simple and fast way, providing the researchers with visual tools that allow to implement case studies expeditiously. The use of this MAS has allowed the balancing and distribution of tasks dynamically in different virtual organizations interacting with each other through FIPA-ACL. The communication protocol used for the interconnection between the different devices is based on the IRC protocol (Internet Relay Chat Protocol-RFC1459), which allows a higher efficiency regarding battery consumption, since it is a lighter and more flexible protocol compared to a SOAP architecture.

To perform the control and act on the physical layer of hardware, a printed circuit control board has been designed and manufactured. This board acts as a gateway between the developed user applications and the wheelchair. This hardware is independent of the wheelchair manufacturers, which allows this solution to be tested on any model and manufacturer. The interface allows different external devices to be connected depending on the degree of user mobility. This case study has been validated with three external interfaces (voice, smartphone and an EEG device). It is important to remark that the development cost of the interface is less than $120, as it was intended at the beginning, so that it could be financed by potential end users. The tests were conducted with the Social Affairs Service of the University of Salamanca (S.A.S). The final application that allows to move a wheelchair through brain stimuli was the interface that attracted the most attention. From a more technical point of view, this control interface allows users to make changes in direction quickly, through visual support or through abstract concepts, with an initial delay of 2 s and sampling every 0.5 s. To calibrate the interface, it is enough to do a small training of 40 s of duration, which allows the platform to be much more efficient and to present satisfactory results. Finally, we could verify that the current state of this work does not allow a user to move inside a closed space with reduced dimensions, rather it intends to be a proof of concept that allows a person suffering from some type of motor problem to move in an open space.

Having not opted for an RSF for the development of the platform might have slowed down the development of the platform, since the MAS development platforms do not provide certain tools that RSF provide and could have been very useful in this work, such as robot interfaces and drivers, robotics algorithms or simulation and modelling tools. However, once the platform has been implemented and deployed, we consider that the use of VO speeds up and improves the incorporation of new control interfaces that combine the use of sensors and artificial intelligence techniques. Many are the advantages of using VO, which allow to achieve more open and dynamic systems, gaining in flexibility, dynamism and openness, while maintaining the implicit advantages of using MAS, such as the system’s heterogeneity, modularity, scalability, parallelism and flexibility capabilities or its robustness against faults.

Despite of the fact that the platform can be easily implemented in any conventional wheelchair, the emergence of initiatives such as the one presented in this work will not reach relevance enough if no impetus is given by wheelchair manufacturers. Nevertheless, this work can be very useful as a starting point for those researchers who require to modify of a conventional wheelchair to carry out their work. However, it will always require an additional effort at hardware level if there is no consensus in the standardization of the protocols with which to interact and control wheelchairs by all manufacturers.

Overall, we are satisfied with the results obtained in terms of the performance of the control methods. However, improvements can still be made at a general level. For example, the incorporation of more advanced systems in the collision system, such as the one proposed in [9] can be used, so that the experience of user control with any interface can be greatly improved. On the other hand, the evaluation of the results obtained could be more exhaustive in terms of control methods by increasing the number of attempts and users but fundamentally incorporating in the case studies people with different disabilities to assess the adequacy of each one of the proposals to each of the disabilities.

## Figures and Tables

**Table 1:** Commercial proportional control systems.

Type of Control	Model	Reference
Joystick	Mo-Vis P002-71	[14]
Sensitive joystick	Invacare LiNX, Ottobock joystick mini abatible	[15,16]
Sealed Joysticks	Permobil Mini Joystick	[17]
Touch pad	Switch-it Touch Drive 2	[18]
Tilt Sensor	Magitek iZIP	[19]

**Table 2:** Commercial non-proportional control systems.

Type of Control	Model	Reference
Reinforced joysticks	Switch-it Tough Joystick (TJS)	[18]
Sensitive mini-joysticks	Invacare Tash Mini Joystick	[15]
Switch trays	Switch-it lap trays	[18]
Single button systems	Invacare Single Switch Scanner	[15]
Proximity sensors	Invacare ASL proximity switch array, Permobil Head Array	[15,17]
Sip & puff	Invacare Sip N’ Puff (SNPM6)	[15]

**Table 3:** Voice commands and system response.

Command	System Response
“Run forward”	The wheelchair moves forward
“Run back”	The wheelchair moves back
“Run left”	The wheelchair moves to the left
“Run right”	The wheelchair moves to the right
“Execute speed (number from 1 to 5)”	The wheelchair’s speed changes (intensity levels from 1 to 5)
“Abort”	Makes the wheelchair stop
“Execute news”	The system reads a news item from the RSS of a newspaper to be defined by the user
“Execute weather”	The system reads the weather forecast
“Execute lights”	Makes the wheelchair lighting system turn on (or off)
“Execute time”	The system reads the current time

**Table 4:** Comparison of models with abstract thoughts.

**Algorithm**	**User 1**			**User 2**			**User 3**		
	**Accuracy (%)**	**Kappa**	**Time (s)**	**Accuracy (%)**	**Kappa**	**Time (s)**	**Accuracy (%)**	**Kappa**	**Time (s)**
RIPPER	97.25	0.9678	1.26	97.16	0.9654	1	97.36	0.9542	1.09
C4.5	98.35	0.9721	0.62	98.23	0.9782	0.27	98.48	0.9624	0.27
R.Forest	96.78	0.9534	0.6	96.42	0.9574	0.5	96.98	0.9408	0.44
Bayes Net	92.56	0.9178	0.7	91.42	0.9107	0.38	92.35	0.9004	0.39
SVM	96.63	0.9578	0.78	96.23	0.9503	0.47	97.87	0.9543	0.45
Bagging	97.84	0.9724	4.53	97.45	0.9549	4.45	97.94	0.9664	4.34
Boosting	97.63	0.9706	0.46	97.59	0.9619	0.46	97.81	0.9546	0.45
**Algorithm**	**User 4**			**User 5**			**Mean**		
	**Accuracy (%)**	**Kappa**	**Time (s)**	**Accuracy (%)**	**Kappa**	**Time (s)**	**Accuracy (%)**	**Kappa**	**Time (s)**
RIPPER	97.59	0.9641	1.04	97.21	0.9601	1.14	97.314	0.9623	1.106
C4.5	97.85	0.9528	0.24	97.45	0.9578	0.24	98.072	0.9647	0.328
R.Forest	97.68	0.9606	0.41	98.46	0.9687	0.45	97.264	0.9562	0.48
Bayes Net	93.21	0.9154	0.35	92.45	0.9124	0.4	92.398	0.9114	0.444
SVM	97.24	0.9629	0.46	97.48	0.9621	0.48	97.09	0.9575	0.528
Bagging	97.69	0.9579	4.15	97.89	0.9578	4.32	97.762	0.9619	4.358
Boosting	98.04	0.9723	0.42	98.12	0.9724	0.44	97.838	0.9664	0.446

**Table 5:** Comparison of models when viewing images.

**Algorithm**	**User 1**			**User 2**			**User 3**		
	**Accuracy (%)**	**Kappa**	**Time (s)**	**Accuracy (%)**	**Kappa**	**Time (s)**	**Accuracy (%)**	**Kappa**	**Time (s)**
RIPPER	98.56	0.9824	0.95	99.3878	0.9923	1.19	98.67	0.9712	1.08
C4.5	98.72	0.9715	0.34	98.52	0.9705	0.31	97.87	0.9641	0.42
R.Forest	95.32	0.9414	0.49	95.12	0.9475	0.51	94.45	0.9376	0.48
Bayes Net	93.78	0.9124	0.4	92.66	0.9122	0.39	91.78	0.9117	0.4
SVM	96.47	0.9612	0.42	96.19	0.9545	0.51	96.36	0.9578	0.46
Bagging	97.12	0.9664	4.49	97.35	0.9642	4.8	96.96	0.9624	4.7
Boosting	96.89	0.9612	0.45	96.94	0.9623	0.48	96.57	0.9612	0.49
**Algorithm**	**User 4**			**User 5**			**Mean**		
	**Accuracy (%)**	**Kappa**	**Time (s)**	**Accuracy (%)**	**Kappa**	**Time (s)**	**Accuracy (%)**	**Kappa**	**Time (s)**
RIPPER	98.87	0.9787	0.96	98.01	0.9784	1.44	98.73445	0.9806	1.124
C4.5	98.84	0.9823	0.34	99.85	0.9895	0.55	98.77	0.9756	0.392
R.Forest	96.06	0.9542	0.5	96.14	0.9581	0.52	95.418	0.9478	0.5
Bayes Net	92.64	0.9228	0.4	94.83	0.9407	0.87	93.138	0.9199	0.492
SVM	96.48	0.9569	0.78	96.12	0.9574	0.48	96.324	0.9576	0.53
Bagging	97.87	0.9735	5.16	97.45	0.9682	4.91	97.35	0.9669	4.812
Boosting	96.49	0.9637	0.52	96.42	0.9578	0.5	96.662	0.9613	0.488

**Table 6:** Evaluation of the control systems (ease of use).

User	Accelerometer		Touchscreen		Voice		EEG	
	Control Accuracy	Ease of Use	Control Accuracy	Ease of Use	Control Accuracy	Ease of Use	Control Accuracy	Ease of Use
User 1	10	10	10	9	**7**	**9**	**7**	**9**
User 2	10	9	9	8	8	8	6	8
User 3	10	10	9	8	8	9	6	7
User 4	10	9	10	9	8	8	5	8
User 5	10	9	10	9	6	8	6	7
Mean	10	9.4	9.4	8.6	7.4	8.4	6	7.8

**Figure A1:**
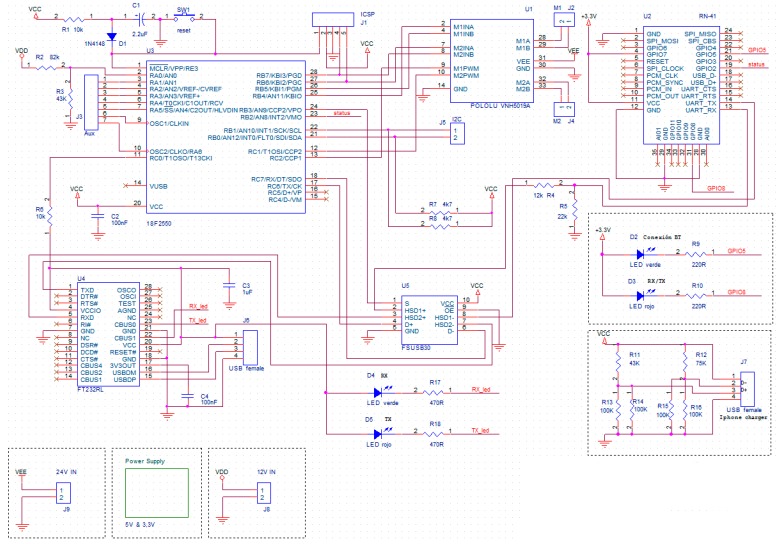
Electric scheme.

## Appendix A

Since other researchers may decide to use the hardware for their own experimentation, we decided to add electrical diagrams and the interconnection of the components designed for this case study in Figure A1.

## References

[1] World Health Organization (2010). Fact Sheet on Wheelchairs.

[2] European Comission (2015). The 2015 Ageing Report.

[3] United Nations (1994). Standard Rules on the Equalization of Opportunities for Persons with Disabilities.

[4] European Comission (2010). European Disability Strategy 2010–2020: A Renewed Commitment to a Barrier-Free Europe.

[5] Schumer C.E. (2015). The Disability Integration Act.

[6] Bonarini A., Ceriani S., Fontana G., Matteucci M. (2013). On the development of a multi-modal autonomous wheelchair. Handbook of Research on ICTs for Human-Centered Healthcare and Social Care Services.

[7] Quaglia G., Nisi M. (2017). Design of a self-leveling cam mechanism for a stair climbing wheelchair. Mech. Mach. Theory.

[8] Ponce P., Molina A., Mendoza R., Ruiz M.A., Monnard D.G., del Campo L.D.F. (2010). Intelligent Wheelchair and Virtual Training by LabVIEW.

[9] Rojas M., Ponce P., Molina A. (2018). A fuzzy logic navigation controller implemented in hardware for an electric wheelchair. Int. J. Adv. Robot. Syst..

[10] Heitmann J., Kohn C., Stefanov D. Robotic wheelchair control interface based on headrest pressure measurement. Proceedings of the 2011 IEEE International Conference on Rehabilitation Robotics.

[11] Kundu S., Mazumder O., Lenka P.K., Bhaumik S. (2017). Hand Gesture Recognition Based Omnidirectional Wheelchair Control Using IMU and EMG Sensors. J. Intell. Robot. Syst..

[12] Fehr L., Langbein W.E., Skaar S.B. (2000). Adequacy of power wheelchair control interfaces for persons with severe disabilities: A clinical survey. J. Rehabil. Res. Dev..

[13] Dicianno B.E., Cooper R.A., Coltellaro J. (2010). Joystick Control for Powered Mobility: Current State of Technology and Future Directions. Phys. Med. Rehabil. Clin. N. Am..

[14] Mo-Vis mo-Vis All-Round Joystick P002-71. http://www.mo-vis.com/en/products/mo-vis-products/all-round-joystick.

[15] Invacare Invacare Field Reference Guide. https://www.invacare.com/doc_files/1141471.pdf.

[16] Ottobock Ottobock Joystick Mini Abatible. http://www.ottobock.es/movilidad/sillas-de-ruedas-electronicas-y-mandos-especiales/mandos-especiales/.

[17] Drive Controls-Permobil. https://permobilus.com/products/power-wheelchairs-by-permobil/accessories/drive-controls/.

[18] Switch-It catalogue. http://www.sunrisemedical.com/getattachment/956dd894-d6ec-4769-9f23-20593e0ad524/Switch-It-Alternative-Drive-Controls-Catalog.aspx.

[19] Magitek Magitek-Products. http://www.magitek.com/products/.

[20] Gundogdu K., Bayrakdar S., Yucedag I. (2017). Developing and modeling of voice control system for prosthetic robot arm in medical systems. J. King Saud Univ. Comput. Inf. Sci..

[21] Rogowski (2012). Industrially oriented voice control system. Robot. Comput. Integr. Manuf..

[22] Zhu J., Gao X., Yang Y., Li H., Ai Z., Cui X. Developing a voice control system for ZigBee-based home automation networks. Proceedings of the 2nd IEEE International Conference on Network Infrastructure and Digital Content.

[23] Škraba A., Stojanović R., Zupan A., Koložvari A., Kofjač D. (2015). Speech-controlled cloud-based wheelchair platform for disabled persons. Microprocess. Microsyst..

[24] Ruíz-Serrano A., Posada-Gómez R., Sibaja A.M., Rodríguez G.A., Gonzalez-Sanchez B.E., Sandoval-Gonzalez O.O. (2013). Development of a dual control system applied to a smart wheelchair, using magnetic and speech control. Procedia Technol..

[25] Peixoto N., Nik H.G., Charkhkar H. (2013). Voice controlled wheelchairs: Fine control by humming. Comput. Methods Programs Biomed..

[26] Leo M., Medioni G., Trivedi M., Kanade T., Farinella G.M. (2017). Computer vision for assistive technologies. Comput. Vis. Image Underst..

[27] Gajwani P.S., Chhabria S.A. (2010). Eye motion tracking for wheelchair control. Int. J. Inf. Technol..

[28] Purwanto D., Mardiyanto R., Arai K. (2009). Electric wheelchair control with gaze direction and eye blinking. Artif. Life Robot..

[29] Leishman F., Horn O., Bourhis G. (2010). Smart wheelchair control through a deictic approach. Rob. Auton. Syst..

[30] Leishman F., Monfort V., Horn O., Bourhis G. (2014). Driving Assistance by Deictic Control for a Smart Wheelchair: The Assessment Issue. IEEE Trans. Hum. Mach. Syst..

[31] Herweg, Gutzeit J., Kleih S., Kübler A. (2016). Wheelchair control by elderly participants in a virtual environment with a brain-computer interface (BCI) and tactile stimulation. Biol. Psychol..

[32] Craig D.A., Nguyen H.T. Adaptive EEG thought pattern classifier for advanced wheelchair control. Proceedings of the 29th Annual International Conference of the IEEE on Engineering in Medicine and Biology Society (EMBS).

[33] Zhang Y., Feng X., Luo Y. (2015). Intelligent wheelchair system based on sEMG and head gesture. J. China Univ. Posts Telecommun..

[34] Mishra S., Norton J.J., Lee Y., Lee D.S., Agee N., Chen Y., Chun Y., Yeo W.H. (2017). Soft, conformal bioelectronics for a wireless human-wheelchair interface. Biosens. Bioelectron..

[35] Hashimoto M., Takahashi K., Shimada M. Wheelchair control using an EOG-and EMG-based gesture interface. Proceedings of the AIM 2009. IEEE/ASME International Conference onAdvanced Intelligent Mechatronics.

[36] Lei S., Li Z. (2013). Fusing Visual Tracking and Navigation for autonomous Control of An Intelligent Wheelchair. IFAC Proc. Vol..

[37] Miyamoto S., Koshizen T., Matsumoto T., Kawase H., Higuchi M., Torimoto Y., Uno K., Sato F. (2018). An Application Using a BLE Beacon Model Combined with Fully Autonomous Wheelchair Control.

[38] Yeounggwang J., Hwang J., YiKim E. (2013). An Intelligent Wheelchair Using Situation Awareness and Obstacle Detection. Procedia Soc. Behav. Sci..

[39] Pasteaua F., Narayanan V.K., Babel M., Chaumette F. (2016). A visual servoing approach for autonomous corridor following and doorway passing in a wheelchair. Rob. Auton. Syst..

[40] Iñigo-Blasco P., Diaz-Del-Rio F., Romero-Ternero C., Cagigas-Muñiz D., Vicente-Diaz S. (2012). Robotics software frameworks for multi-agent robotic systems development. Rob. Auton. Syst..

[41] Collett T.H.J., MacDonald B.A., Gerkey B.P. Player 2.0: Toward a practical robot programming framework. Proceedings of the Australasian conference on robotics and automation (ACRA 2005).

[42] Bruyninckx H. Open robot control software: The OROCOS project. Proceedings of the 2001 ICRA. IEEE International Conference on Robotics and Automation.

[43] Quigley M., Conley K., Gerkey B., Faust J., Foote T., Leibs J., Wheeler R., Ng A.Y. ROS: An open-source Robot Operating System. Proceedings of the ICRA Workshop on Open Source Software.

[44] Ando N., Suehiro T., Kotoku T. A software platform for component based rt-system development: Openrtm-aist. Proceedings of the International Conference on Simulation, Modeling, and Programming for Autonomous Robots.

[45] Calisi D., Censi A., Iocchi L., Nardi D. OpenRDK: A modular framework for robotic software development. Proceedings of the IEEE/RSJ International Conference on Intelligent Robots and Systems, IROS 2008.

[46] Cena G., Cardenas P.F., Pazmino R.S., Puglisi L., Santonja R.A. (2013). A cooperative multi-agent robotics system: Design and modelling. Expert Syst. Appl..

[47] Merdan M., Moser T., Vrba P., Biffl S. (2011). Investigating the robustness of re-scheduling policies with multi-agent system simulation. Int. J. Adv. Manuf. Technol..

[48] Marconi L., Melchiorri C., Beetz M., Pangercic D., Siegwart R., Leutenegger S., Carloni R., Stramigioli S., Bruyninckx H., Doherty P. The SHERPA project: Smart collaboration between humans and ground-aerial robots for improving rescuing activities in alpine environments. Proceedings of the 2012 IEEE International Symposium on Safety, Security, and Rescue Robotics (SSRR).

[49] Rubenstein M., Cabrera A., Werfel J., Habibi G., McLurkin J., Nagpal R. Collective transport of complex objects by simple robots: Theory and experiments. Proceedings of the International Conference on Autonomous Agents and Multi-Agent Systems.

[50] Ardiny H., Witwicki S., Mondada F. (2015). Are autonomous mobile robots able to take over construction? A review. Int. J. Robot. Theory Appl..

[51] Saeedi S., Trentini M., Seto M., Li H. (2016). Multiple-Robot Simultaneous Localization and Mapping: A Review. J. F. Robot..

[52] Wang Y., de Silva C.W. (2010). Sequential Q-Learning with Kalman Filtering for Multirobot Cooperative Transportation. IEEE/ASME Trans. Mechatron..

[53] Bellifemine F., Poggi A., Rimassa G. (1999). JADE—A FIPA-Compliant Agent Framework.

[54] Luke S., Cioffi-Revilla C., Panait L., Sullivan K. Mason: A new multi-agent simulation toolkit. Proceedings of the 2004 Swarmfest Workshop.

[55] Zato C., Villarrubia G., Sánchez A., Barri I., Rubión E., Fernández A., Rebate C., Cabo J.A., Álamos T., Sanz J. (2012). PANGEA—Platform for Automatic coNstruction of orGanizations of intElligent Agents.

[56] Wang J., Chen W., Liao W. (2013). An improved localization and navigation method for intelligent wheelchair in narrow and crowded environments. IFAC Proc. Vol..

[57] Berjón R., Mateos M., Muriel I., Villarrubia G. (2012). Automatic Route Playback for Powered Wheelchairs. Highlights on Practical Applications of Agents and Multi-Agent Systems.

[58] Kumar S., Kumar S. (2015). Design and development of head motion controlled wheelchair. Int. J. Adv. Eng. Technol..

[59] Leeb R., Friedman D., Müller-Putz G.R., Scherer R., Slater M., Pfurtscheller G. (2007). Self-paced (asynchronous) BCI control of a wheelchair in virtual environments: A case study with a tetraplegic. Comput. Intell. Neurosci..

[60] Galán F., Nuttin M., Lew E., Ferrez P.W., Vanacker G., Philips J., Millán J.D. (2008). A brain-actuated wheelchair: Asynchronous and non-invasive brain--computer interfaces for continuous control of robots. Clin. Neurophysiol..

[61] Google Consumer Barometer. https://www.consumerbarometer.com/en/.

[62] Finger Steering Control DX-RJM-VIC MANUAL. https://dynamiccontrols.com/en/downloads/dx/obsolete-dx-product/65-finger-joystick-installation-manual/file.

[63] Fürnkranz J., Widmer G. (1994). Incremental reduced error pruning. Proceedings of the Machine Learning.

[64] Cohen W.W. (1995). Fast effective rule induction. Proceedings of the Machine Learning.

[65] John R. (1993). Quinlan, C4.5: Programs for Machine Learning.

[66] Breiman L. (2001). Random Forests. Mach. Learn..

[67] Su J., Zhang H. Full Bayesian network classifiers. Proceedings of the 23rd international conference on Machine learning.

[68] Vapnik V.N. (1999). An overview of statistical learning theory. IEEE Trans. Neural Netw..

[69] Breiman L. (1996). Bagging Predictors. Mach. Learn..

[70] Schapire R.E., Freund Y. (2012). Boosting: Foundations and Algorithms.

[71] Frank M.H.E., Holmes G., Reutemann B.P.P., Witten I.H. The WEKA Data Mining Software: An Update. https://www.cs.waikato.ac.nz/~eibe/pubs/weka_update.pdf.

